# Unusual Presentation of Papillary Microcarcinoma of Thyroid as Thigh Mass

**DOI:** 10.1155/2011/651701

**Published:** 2011-09-07

**Authors:** Koyye Ravindranath Tagore, S. Ramineni Asok Kumar

**Affiliations:** Department of Pathology, MNR Medical College, Narasapur Road, Andhra Pradesh, Sangareddy, India

## Abstract

An elderly otherwise healthy male presented with a mass in thigh. Fine needle aspiration cytology (FNAC) revealed features of papillary carcinoma of thyroid, suggesting secondary deposits. Later the patient was clinically evaluated. There was no obvious thyromegaly both clinically and sonologically. The patient was followed up. One month after initial presentation, he came with an enlarged cervical lymph node and a tiny nodule in thyroid. FNAC from both the thyroid and cervical lymph node showed identical features of papillary carcinoma.

## 1. Introduction

Papillary thyroid carcinomas are the most common type of differentiated thyroid carcinoma. The majority of these lesions are small; those diameters measuring ≤1 cm have been designated by the World Health Organization as papillary microcarcinoma of the thyroid (PMCT). In the older literature, they were frequently referred to as occult thyroid cancers. 

PMCT usually presents as latent (found incidentally) or as an occult tumor (found after detection of metastasis). Thyroid microcarcinoma is most often papillary, 65%–99% of the cases [1, 2]. The mean age at diagnosis of patients with thyroid microcarcinoma has been reported by different studies to be 41.9 to 55 years [1]. 

Most of the studies revealed that PMCT, more common in women and male-female ratios is of 1 : 4. In a single study describing 141 cases of PMCT <10 mm in diameter, 82 (58%) were men and 59 (42%) were women [3]. It is likely that the higher prevalence of thyroid microcarcinoma in living women may be due to their higher prevalence of thyroid disease and, therefore, greater access to diagnostic procedures resulting in increased identification of PMCT. Papillary thyroid carcinomas are slow-growing tumours more frequently metastases to lymph nodes, less in the lung and bone. PMCT with diffuse metastases to the skin, thigh muscle, larynx, hypopharynx, and breast are unusual [4–6]. In the present case the tumor initially presented as a thigh mass.

## 2. Case Report

**Figure 1:**
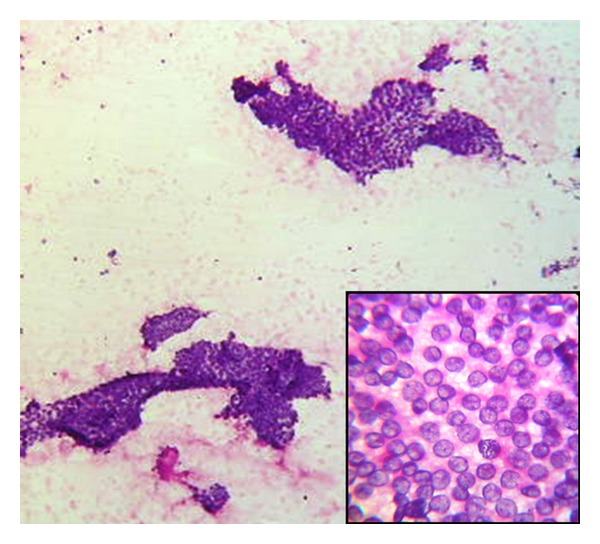
FNAC from thigh swelling shows monolayered sheets and papillary structures of follicular cells. Background shows thick colloid. Inset show intranuclear grooves and nuclear cytoplasmic vacuoles.

**Figure 2:**
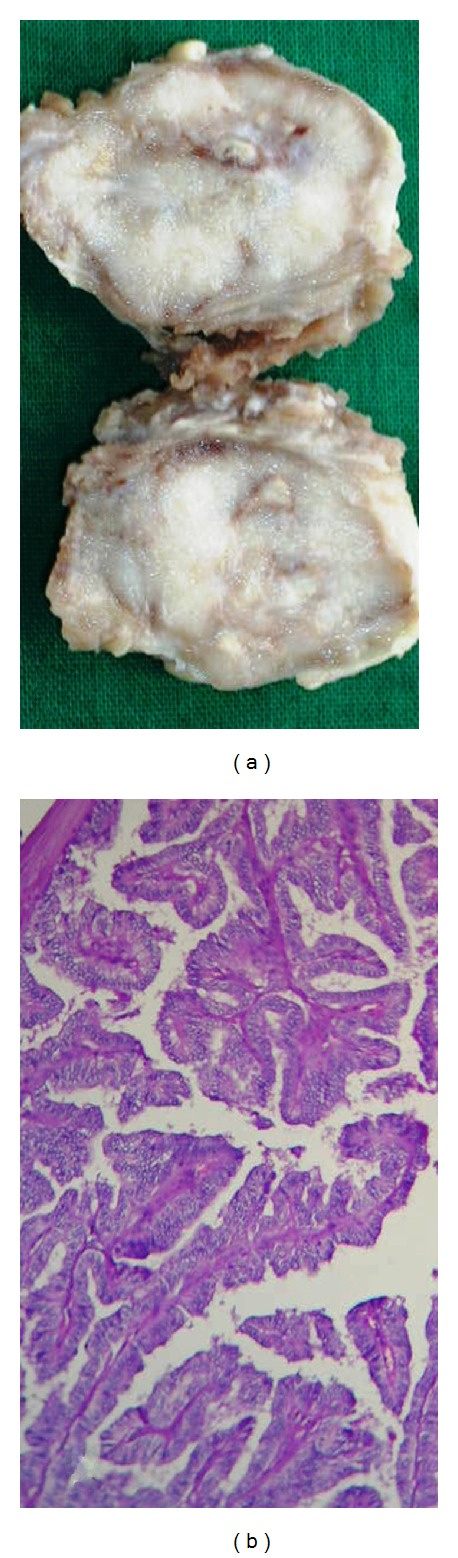
(a) Nodule excised from the thigh showing gray white solid areas with papillary excrescences. (b) Sections from the thigh nodule show papillary structures with ground glass nuclei 40x.

A 55-years-old male, with no significant past medical history, presented with a swelling in left thigh of two-month duration that was gradually increasing in size. The swelling was 4 × 3 cm hard mass over anterior aspect of left thigh that showed restricted mobility. Systemic examination was noncontributory. There was no organomegaly and no other swellings anywhere. FNAC was performed on thigh swelling. The yield was rich and consisted of epithelial cells that were arranged in sheets and well-formed papillary configurations (Figure 1). There were intra nuclear cytoplasmic inclusions and nuclear grooves (Figure 1 inset). Based on the above findings excision biopsy of the mass was advised to confirm papillary thyroid carcinomatous secondary deposits (Figures 2(a) and 2(b)). 

The case was followed up. One month later, the patient came with a small left cervical lymph node and a vague nodule in the thyroid. Ultrasonography of thyroid showed a hypoechoic nodule of 7 × 8 mm in size with areas of punctuate calcification in the left lobe of thyroid. The outlines of the nodule were ill defined and irregular. FNAC of both cervical lymph node and thyroid nodule and excision biopsy microscopically showed the picture of papillary carcinoma thyroid. Metastatic thigh mass on immunocytochemical staining for thyroglobulin yielded positive cytoplasmic reaction in the neoplastic cells.

## 3. Discussion

Papillary microcarcinomas are incidental findings in 25%–36% of thyroidectomy samples done for other reasons and in population-based autopsy studies [7]. They usually are associated with excellent prognosis even if metastasized to lymph nodes or to distant sites. Roti et al. observed in their study that lymph node metastases are more frequent in patients with larger PMCT, >5 mm in size [8]. Lymph node metastases and extrathyroid extension is observed in only 4.4% and 25.7%, respectively, of patients with PMCT ≤5 mm in diameter [9]. In our present case the nodule is 8mm in size and presented with both nodal and distant metastases.

Distant metastases at diagnosis are a rare event. Therefore, only few studies have statistically analyzed possible risk factors. Distant metastases at diagnosis correlated positively with the diameter of PTMC (*P* ≤ 0.05) [8], advancing age (*P* ≤ 0.01), lymph-node metastasis at diagnosis (*P* < 0.01), and follicular variant of PTMC (*P* < 0.008) [10]. In one study it was observed that all patients with distant metastases had lymph-node invasion at diagnosis [7]. The reported common metastatic sites for PTMC were cervical lymph nodes, submandibular salivary glands, lungs, skeleton, and the brain. Diffuse metastases to the skin, thigh muscle, larynx, hypopharynx, and breast are unusual [4–6]. This case is being presented for its exceptional rarity of an occult thyroid papillary carcinoma with distant metastases in unusual locations, like thigh.
